# Supplementary material for: Influenza Virus Membrane Fusion Is Promoted by the Endosome-Resident Phospholipid Bis(monoacylglycero)phosphate
Source: J Phys Chem B. 2022 Dec 5;126(49):10445–51. doi: 10.1021/acs.jpcb.2c06642 (PMC9761668; doi:10.1021/acs.jpcb.2c06642)
Supplement: Supplementary file 1 — jp2c06642_si_001.pdf [file jp2c06642_si_001.pdf]

Supporting Information for

Influenza Virus Membrane Fusion is Promoted by the Endosome-Resident Phospholipid Bis(monoacylglycero)phosphate

Steinar Mannsverk[1], Ana M. Villamil Giraldo[1], and Peter M. Kasson[1,2,*]

[1]Science for Life Laboratory, Department of Cell and Molecular Biology, Uppsala University, Uppsala 75124 Sweden and [2]Departments of Molecular Physiology and Biomedical Engineering, University of Virginia, Charlottesville VA 22908.
*to whom correspondence should be addressed. kassonlab@gmail.com

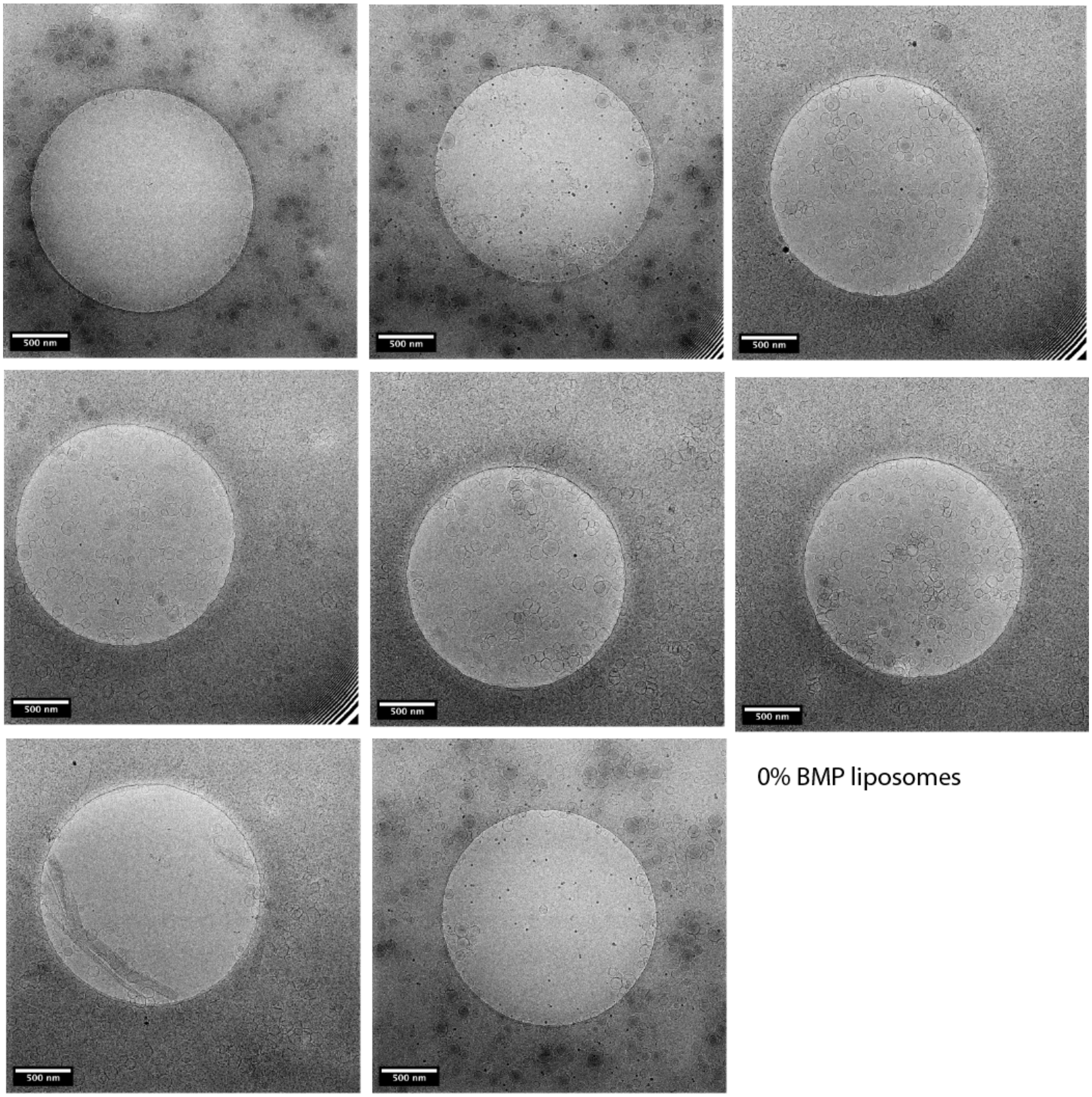

0% BMP liposomes

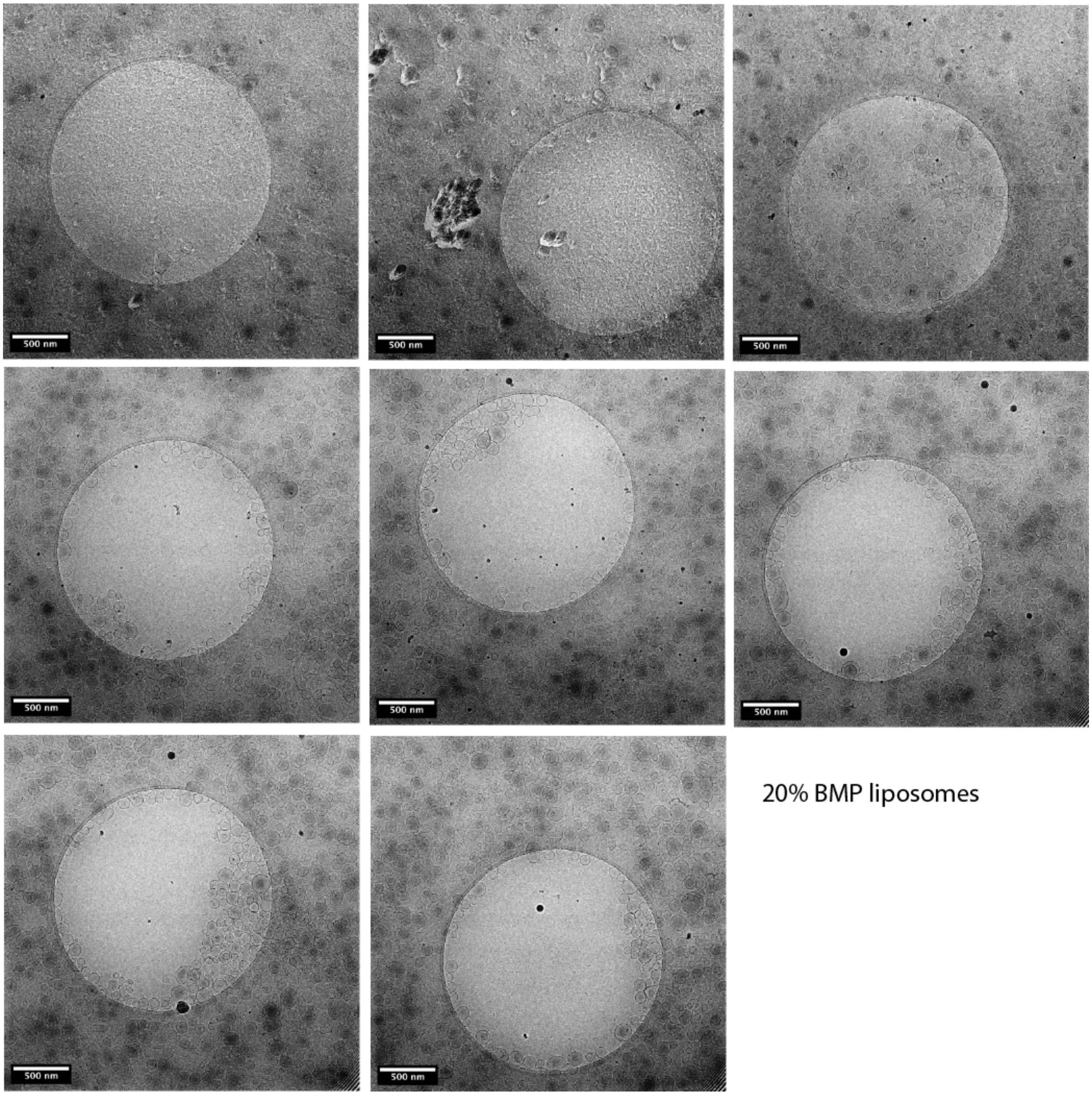

Figure S1. Images of all electron micrographs acquired. Thumbnail images of electron micrographs acquired are displayed; 8 and 8 images representing liposomes containing 0 mol % and 20 mol % BMP, respectively.

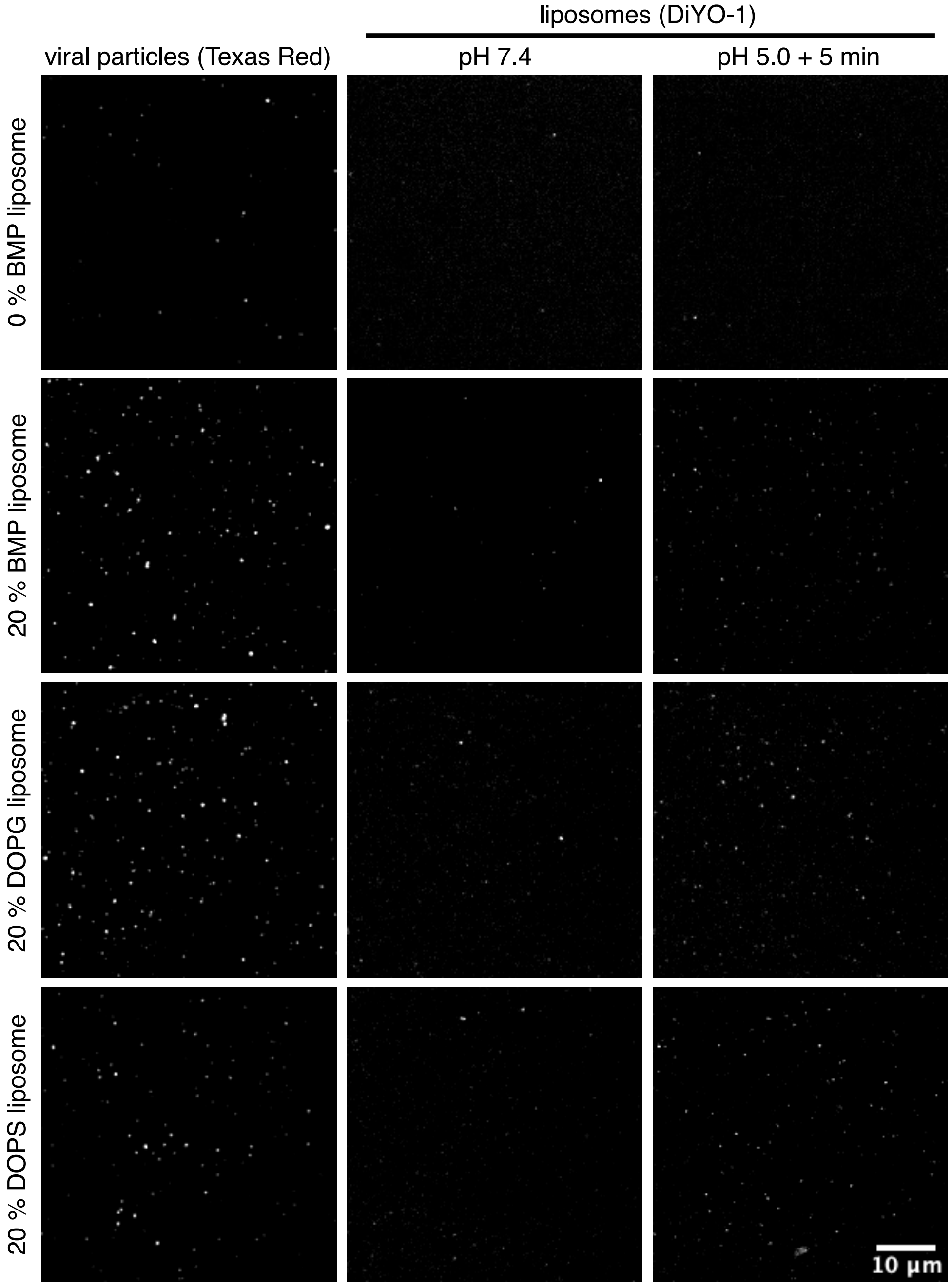

Figure S2. Additional micrographs of content mixing events with liposomes containing anionic phospholipids. Fluorescence micrographs shows viral particles undergoing content mixing with target liposomes containing 0 mol % BMP (first row), 20 mol % BMP (second row), 20 mol % DOPG (third row) or 20 mol % DOPS (fourth row). "Viral particles" (first column) displays membrane-labelled viral particles and "liposomes" displays liposomes before (second column) and 5 min after the pH drop (third column). White spots visualized after but not before pH drop represent liposomes that have undergone content mixing with virus. A small number of liposomes displayed DiYO-1 fluorescence prior to the pH drop (second column). Scale bar displayed in last image applies to all images.

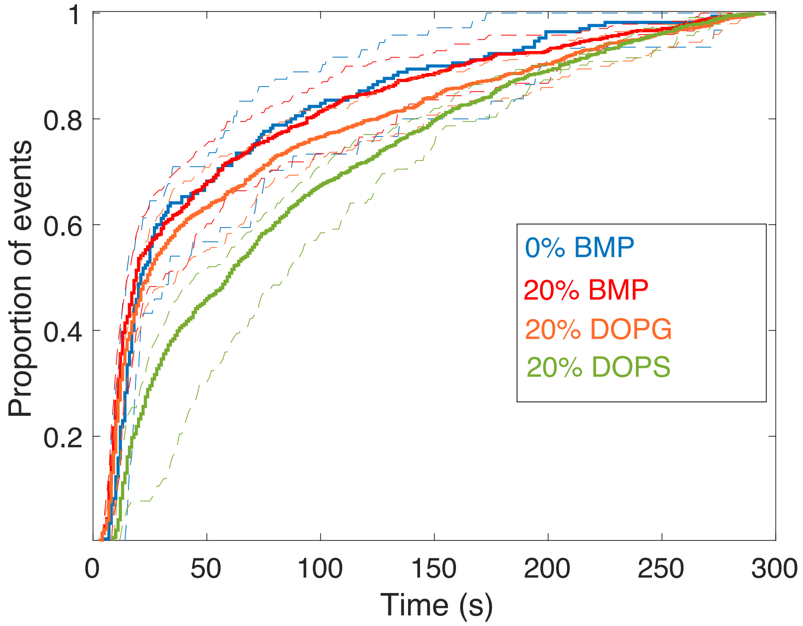

Figure S3. Uncertainty analysis of content mixing kinetics. Uncertainty distributions were quantified for content mixing experiments via bootstrap resampling across flow cell channels (experimental replicates). Cumulative distributions are plotted with the observed cumulative distribution function in solid lines and the 90% confidence intervals in dashed lines. Distributions were assessed as different or not via bootstrapped rank sum tests of individual virus waiting times, bootstrap resampled across flow cell channels.
